# Video Motion Analysis as a Quantitative Evaluation Tool for Essential Tremor during Magnetic Resonance-Guided Focused Ultrasound Thalamotomy

**DOI:** 10.3390/neurolint15040091

**Published:** 2023-11-29

**Authors:** Mayumi Kaburagi, Futaba Maki, Sakae Hino, Masayuki Nakano, Toshio Yamaguchi, Masahito Takasaki, Hirokazu Iwamuro, Ken Iijima, Jinichi Sasanuma, Kazuo Watanabe, Yasuhiro Hasegawa, Yoshihisa Yamano

**Affiliations:** 1 Department of Neurology, St. Marianna University School of Medicine, Kanagawa 216-8511, Japan; mayumi.hasegawa@marianna-u.ac.jp (M.K.); f2maki@marianna-u.ac.jp (F.M.); hasegawa-neuro1@marianna-u.ac.jp (Y.H.); 2 Department of Neurology, Shin-Yurigaoka General Hospital, Kanagawa 215-0026, Japan; 3 Department of Neurosurgery, Shin-Yurigaoka General Hospital, Kanagawa 215-0026, Japan; mn.jn.yn.yn@gmail.com (M.N.);; 4 Research Institute for Diagnostic Radiology, Shin-Yurigaoka General Hospital, Kanagawa 215-0026, Japan; 5 International Academia for Focused Ultrasound Therapy, Kanagawa 215-0023, Japan; 6 Department of Anesthesiology, Shin-Yurigaoka General Hospital, Kanagawa 215-0026, Japan; 7 Department of Neurosurgery, Juntendo University, Tokyo 113-8421, Japan; 8 Department of Diagnostic Radiology, Saitama Sekishinkai Hospital, Saitama 350-1305, Japan

**Keywords:** video motion analysis, essential tremor, magnetic resonance-guided focused ultrasound thalamotomy, Clinical Rating Scale for Tremor (CRST)

## Abstract

The Clinical Rating Scale for Tremor (CRST) is commonly used to evaluate essential tremor (ET) during focused ultrasound (FUS) thalamotomy. However, it faces challenges such as the ceiling effect and test–retest variability. This study explored the utility of videographic motion analysis as an evaluation index for ET. Forty-three patients with ET performed postural tremor and line-drawing tasks recorded on video, and the data were analyzed using motion analysis software. The test–retest and inter-rater reliability, correlations with the CRST and tremor scores, and pre/post-FUS treatment comparisons were analyzed. The video motion analysis showed excellent test–retest and inter-rater reliability. In the postural tremor tasks, video parameter amplitude significantly correlated with the CRST and tremor scores. Similarly, for the line-drawing task, video parameter amplitude showed significant correlations with CRST and tremor scores, effectively addressing the ceiling effect. Regarding post-FUS treatment improvements, changes in the CRST and tremor scores were significantly associated with changes in video parameter amplitude. In conclusion, quantitative analysis of the video motion of ET enables precise evaluation of kinematic characteristics and effectively resolves the ceiling effect and test–retest variability. The video motion analysis score accurately reflected the tremor severity and treatment effects, demonstrating its high clinical utility.

## 1. Introduction

Essential tremor (ET) is a prevalent neurological disorder, affecting approximately 2.5–4% of adults, significantly impairing daily activities in severe cases [1]. Despite pharmacological interventions being the primary treatment, almost half of patients exhibit resistance to these therapies [2,3]. Consequently, magnetic resonance-guided focused ultrasound (MRgFUS) thalamotomy has emerged as a prominent alternative for medication-resistant ET [4]. MRgFUS thalamotomy is a minimally invasive treatment using focused ultrasound to ablate the target tissue thermally. During the procedure, the physician interacts with the patient, evaluates the effectiveness of the treatment for tremor, and administers the treatment while ensuring safety and efficacy. Therefore, improving the accuracy of evaluating treatment effectiveness during the procedure is essential for enhancing the efficacy and safety of the treatment.

The Fahn–Tolosa–Martin Clinical Rating Scale for Tremor (CRST) [5] (Supplementary Figure S1), which has been internationally standardized and validated, is traditionally used as a clinical assessment tool for evaluating symptoms reported by patients with ET during MRgFUS. This rating scale evaluates the severity of tremor in the limbs during rest, posture, and action based on the amplitude of the tremor through visual assessment by the rater. Consequently, obtaining reliable scores requires experienced raters, and because the amplitude of tremor in patients undergoing surgical treatment can exceed the cutoff value of 4 cm, which corresponds to an evaluation score of four (“severe tremor”), it is necessary to consider the ceiling effect. Thus, its reliance on visual assessment by raters and the inherent ceiling effect signal the need for more precise and quantitative methods. Recently, wearable 3D accelerometers have been explored to overcome these limitations, offering continuous numerical tremor measurements [6,7,8,9].

Building on this, our study explores the potential of an alternative approach—video motion analysis using the quantitative 2D motion analysis software DIPP-Motion V/2D^®^ (DITECT Corporation, Tokyo, Japan). This technology allows for the quantitative analysis of tremors by marking areas of interest on markerless recorded videos, utilizing advanced image processing. We hypothesize that this method could offer a more nuanced assessment of tremor severity, which would be particularly useful during MRgFUS thalamotomy where precise tremor evaluation is critical.

This study aims to evaluate the effectiveness of video motion analysis in patients undergoing MRgFUS thalamotomy. By comparing the video motion analysis data with traditional CRST scores before and after the procedure, we aim to assess the method’s reproducibility and its correlation with established clinical metrics.

## 2. Materials and Methods

### 2.1. Study Population

The study population comprised 43 consecutive patients with medication-resistant ET who underwent MRgFUS thalamotomy targeting the ventral intermediate nucleus (Vim) at Shin-Yurigaoka General Hospital between July 2020 and October 2021. Video recordings were performed before and after MRgFUS thalamotomy, and tremor severity was evaluated using the CRST. Medication-resistant tremor was defined as tremor where medication remained ineffective despite using at least two different drugs at the maximum tolerated dose [10]. MRgFUS thalamotomy was indicated for patients with severe tremors (with a score of 2 or more on any item of the CRST). Patients with neurodegenerative diseases, unstable cardiac conditions, or coagulation abnormalities were excluded. The exclusion criteria did not include a cutoff value for skull density ratio (SDR). This study was conducted at a single hospital and approved by the Ethics Review Board of Shin-Yurigaoka General Hospital (Approval No. 20220822-2). Informed consent was obtained from all patients prior to treatment.

### 2.2. MRgFUS Thalamotomy Procedure

The MRgFUS thalamotomy was performed according to a previously reported protocol [11]. Briefly, the patient’s hair was shaved, and a stereotactic frame was attached under local anesthesia. All patients underwent MRgFUS treatment using the ExAblate Neuro system (Insightec, Tirat Carmel, Israel) and a 3.0-T (DV25) Discovery MR750w scanner (GE Healthcare, Japan). In all cases, the target was the Vim on one side. Therapeutic ultrasound ablation was performed stereotactically using up to 1024 transducers to create a three-dimensional coagulation lesion. The temperature at the ablation site was monitored using MR thermometry, and the optimal temperature for ablation was between 55 °C and 60 °C. Ultrasound ablation was performed while continuously monitoring the patient, and the procedure was repeated until tremor relief was observed.

### 2.3. Clinical Rating Scale for Tremor

The clinical severity of the tremor was evaluated using the CRST [4,12,13,14,15,16,17,18] (Supplementary Figure S1). The CRST consists of three parts: Part A evaluates tremor at rest, posture, and action; Part B evaluates tremor during tasks such as writing, drawing, and pouring; and Part C evaluates functional disabilities in activities of daily living (ADL). All items are scored on a scale of 0 to 4, with higher scores indicating more severe tremor [5]. Tremor scores ranging from 0 to 32, with higher scores indicating more severe tremor, were calculated using values extracted from Part A (rest, posture, and action items) and Part B (writing, drawing, and pouring tasks) of the CRST for both the treated and untreated upper limbs [4]. The assessment of tremor severity in Part A was based on a visual evaluation of tremor amplitude. Specifically, it was defined as follows: 0 = none; 1 = slight, barely perceivable, and may be intermittent; 2 = moderate, amplitude < 2 cm; 3 = marked, amplitude 2–4 cm; and 4 = severe, amplitude > 4 cm. The evaluation of the five tasks in Part B was based on a subjective assessment. For example, the evaluation of the 15 cm line-drawing task, which is one of the tasks in CRST Part B, is defined as follows: 0 = normal; 1 = slightly tremulous, may occasionally cross lines; 2 = moderately tremulous or crosses lines frequently; 3 = accomplishes the task with great difficulty, many errors; and 4 = unable to complete the drawing. The items in CRST Part C evaluate functional disabilities in speaking (C16), eating (C17), drinking (C18), hygiene (C19), dressing (C20), writing (C21), working (C22), and social activities (C23). These items were scored by the patients themselves, except for speaking, which was evaluated by the rater. The clinical severity of the tremor at the wing-beat position was also evaluated using the essential tremor rating assessment scale (TETRAS), which was developed by the Tremor Research Group [19]. In the CRST scale, an upper extremity tremor greater than 4 cm corresponds to a maximum rating of 4, while a grade 4 tremor in TETRAS corresponds to an amplitude greater than 20 cm. Specifically, it was defined as follows: 0 = none; 1 = amplitude < 0.5 cm; 1.5 = 0.5–1.0 cm; 2 = 1.0–< 3.0 cm; 2.5 = 3.0–< 5.0 cm; 3 = 5–< 10.0 cm; 3.5 = 10.0–< 20.0 cm; and 4 = >20.0 cm.

### 2.4. Videographic Analysis

**Figure 1:**
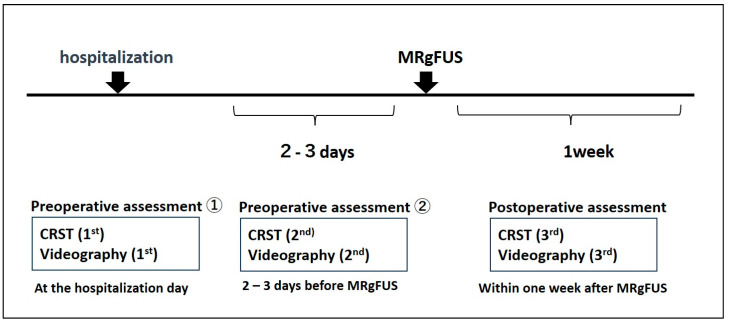
Timeline of Video Recordings and Clinical Evaluations for Tremor Movements Using CRST.

Video recordings of tremor movements and clinical evaluations using CRST were conducted three times: the first preoperative evaluation at the time of hospital admission, the second preoperative evaluation 2–3 days before MRgFUS, and the postoperative evaluation within one week after MRgFUS (Figure 1). The analysis of test–retest reliability was conducted by comparing data from the first and second preoperative evaluations. To assess the effectiveness of MRgFUS, data from the first preoperative evaluation and the postoperative evaluation were compared. Tremor movements during the postural tremor and 15 cm line-drawing tasks in the upper limbs were recorded using a high-definition video imaging device (25 fps, 1280 × 720 px) at a distance of 1.0 m from the patient’s upper limbs. The recorded videos were analyzed using motion analysis software (DIPP-Motion V/2D^®^, DITECT Corporation, Tokyo, Japan) with digitized data.

For the analysis of the postural tremor task (Supplementary Figure S2, Supplementary Movie 1: A normal volunteer demonstrated movements that mimic tremor for illustrative purposes), the “wing-beat” posture was adopted, where the elbow was flexed, the upper limb was held horizontally, and the hand was placed in front of the chest. This posture, established by TETRAS [19], is suitable for detecting tremor in the upper limbs in a two-dimensional plane. The postural tremor task was video-recorded for 60 s, and the video frames of the 10 s period showing the highest amplitude were used for two-dimensional motion analysis. After video recording, three anatomical regions of interest (finger, wrist, and elbow) were manually selected from the video, and their XY coordinate values were automatically tracked for each frame (25 fps). We utilized data from the 5th to the 55th second after the initiation of a 60 s video recording, focusing on the time point with the highest amplitude within ±5 s (a total of 10 s). At the 0 s time point within this 10 s interval, we employed values from the horizontal (*x*-axis) and vertical (*y*-axis) axes relative to the upper-left corner of the screen. Motion parameters such as average speed (mm/s) and average acceleration (mm/s2) were calculated as follows. For instance, if moving from frame 1 (X1, Y1) to frame 2 (X2, Y2), the displacement was calculated using the Pythagorean theorem: Displacement = √(ΔX^2 + ΔY^2). Time was set in *Y*-axis time units, and velocity was calculated using the formula: Velocity = Displacement/Time. Acceleration was determined using the current frame velocity (V1), the previous frame velocity (V0), and the time between frames (t) with the formula: Acceleration = (V1 − V0)/t. The frequency was calculated as the average number of waves per second over the adopted 10 s period, and the amplitude was determined using the geometric mean of amplitude over the 10 s duration.

For the 15 cm line-drawing task (Supplementary Figure S3, Supplementary Movie S2: A normal volunteer demonstrated movements that mimic tremor for illustrative purposes), the patient was instructed to connect the points on the left and the right without resting their hand or arm on the table. This was achieved using the line with the widest gap at the top of the figure for the Drawing C task of CRST PART B (Supplement Figure S1). After the video recording, the target point was set at the tip of the pen. The target was automatically tracked using analysis software, and the XY coordinate values for each frame (25 fps) were calculated. The motion parameters of the tremors, including average speed (mm/s), average acceleration (mm/s^2^), average frequency (Hz), average amplitude (mm), and cumulative length (mm), were computed. The term “cumulative length of the tremor” in this context refers to the total length of movement made by the pen tip during the 15 cm line-drawing task.

Since the severity of tremor is known to be logarithmically related to tremor motion characteristics, specifically the analyzed measurement values of tremor [20,21,22,23], in this study, the measured values of tremor obtained through video analysis before and after MRgFUS thalamotomy (pre-treatment: T1, post-treatment: T2) were logarithmically transformed for statistical analysis. Changes in tremor motion were calculated using logT2/T1.

### 2.5. Statistical Analysis

Test–retest and inter-rater reliabilities were assessed using intraclass correlation coefficients (ICCs) and their corresponding 95% confidence interval (CI). For test–retest reliability, video analyses of the postural tremor and 15 cm line-drawing tasks were performed by a single rater (M.K.) for ten patients on different days at intervals of 1–3 days. Similarly, for inter-rater reliability, three raters (M.K., S.H., and F.M.) independently performed video analyses on ten patients with ET, and the ICCs and corresponding 95% CI were calculated. ICC (1,1) and ICC (2,3) were calculated for the video analysis data of ET, and test–retest and inter-rater reliabilities were analyzed for the ten patients with ET. Based on the ICC values, reliability was categorized as follows: ICC ≥ 0.90: excellent reliability; ICC ≥ 0.70 to <0.90: good reliability; ICC ≥ 0.50 to <0.70: moderate reliability; ICC ≥ 0.30 to <0.50: poor reliability; and ICC < 0.30: very poor reliability. Additionally, the 95% CI of the minimal detectable change (MDC95) was calculated as 1.96 * SDd (SDd is the standard deviation of the differences). To assess the test–retest reliability, the percentage of minimum detectable change (MDC%) and standard deviation of the differences (SDd) of test–retest data for the videographic amplitude values and for CRST Part A scores were analyzed based on the previously reported formula [8]. Since the data were not normally distributed, log10 transformation was performed to normalize these data.

The Wilcoxon signed-rank test was used to evaluate changes in CRST scores, tremor scores, and video analysis parameters before and after MRgFUS thalamotomy. The correlations between CRST total scores and tremor scores, and the logarithm of the amplitude values on video analysis parameters (logT1) before MRgFUS thalamotomy, were examined using Spearman’s rank correlation test.

Tremor scores, CRST Part B scores, and CRST Part C scores before MRgFUS thalamotomy were defined as R1, and the corresponding scores after MRgFUS thalamotomy were defined as R2. The values of videographic parameters before MRgFUS thalamotomy were defined as T1, and the corresponding values after MRgFUS thalamotomy were defined as T2. For the correlations between the improvement in tremor scores (R2-R1) and improvement in the amplitude values on video analysis (logT2/T1) for the postural tremor task and the 15 cm line-drawing task, Spearman’s rank correlation test was used for analysis.

The three anatomical targets in the postural tremor task showed a pattern in which the finger had the largest tremor amplitude, followed by the wrist and elbow; this pattern was consistent across individual patients. Therefore, the video analysis parameters of finger tremor were used in the analysis.

## 3. Results

### 3.1. Patient Demographics

A total of 43 patients with medication-resistant ET underwent MRgFUS treatment. The patient demographics are presented in Table 1. The mean age of the patients was 69.9 ± 11.8 years, with 29 (67.4%) being male. The disease duration was 27.7 ± 18.1 years, and the CRST total score ranged from 10 to 76, representing a wide distribution from mild to severe. To examine the test–retest and inter-rater reliabilities of the video analysis data, the initial ten consecutive patients from the 43 patients were selected. The clinical characteristics of these ten patients did not differ significantly from those of the other patients.

### 3.2. Test–Retest and Inter-Rater Reliabilities of the Videographic Parameters

To assess the reproducibility of the videographic data, test–retest and inter-rater reliabilities were analyzed. The test–retest reliability of the videographic parameters obtained from the postural tremor task and 15 cm line-drawing tasks for the same ten patients on different days is presented in Table 2, showing the ICC (1,1) values and 95% CIs of the MDC95. The inter-rater reliability of these parameters is presented in Table 2, which shows the ICC (2,3) values. The ICC values of the test–retest and inter-rater reliabilities exceeded the threshold of 0.9, indicating excellent reliability of the video-graphic analysis data.

We conducted a more detailed assessment of the reproducibility of amplitude values. The MDC% for the videographic amplitude values obtained from the postural tremor task was 61% of the baseline geometric mean tremor amplitude (Table 3). The MDC%s for CRST Part A and TETRAS scores obtained from the postural tremor task were 89% and 84% of mean baseline, respectively. In the postural tremor task, the SDd for videographic amplitude values obtained was 0.211, while the SDd for CRST Part A and TETRAS were 0.823 and 0.408, respectively. In the 15 cm line-drawing task, the SDd for videographic amplitude values obtained was 0.056, while the SDd for CRST Part A was −0.100 (Table 3).

### 3.3. Correlation between Videographic Parameters and Clinical Rating Scores

To investigate whether the videographic analysis data accurately reflected clinical severity, we examined the correlation between the videographic parameters (logT1) and the CRST total score, CRST Part A, Part B (treatment side), and Part C, and the tremor score in the 43 patients before MRgFUS thalamotomy (Table 4). In the postural task, the video-graphic amplitude values showed significant correlations with the CRST total score, CRST Part A, Part B, and Part C, and the tremor score. In the 15 cm line-drawing task, the videographic tremor amplitude values showed significant correlations with the CRST total score, CRST Part A, Part B, and Part C, and the tremor score. The cumulative length showed significant correlations with the CRST total score, CRST Part B, and Part C, and the tremor score.

### 3.4. Videographic Parameters as an Assessment Tool to Evaluate Activities of Daily Living

**Figure 2:**
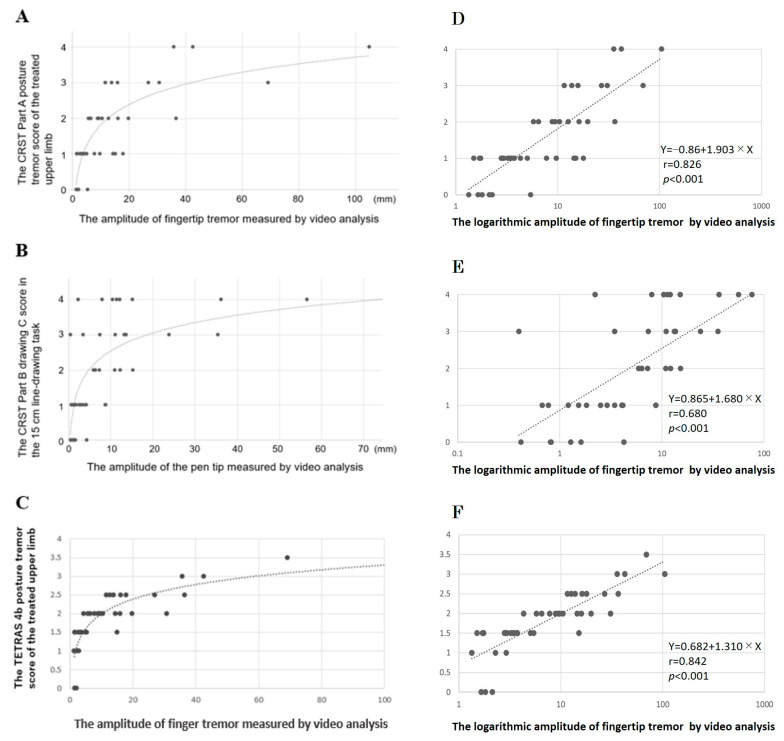
Reducing the Ceiling Effect in CRST and TETRAS Evaluations Using Video Analysis. (**A**) Relationship between CRST Part A scores and the amplitude of videographic parameters during the postural tremor task. The CRST Part A scores are plotted on the vertical axis, with videographic amplitude on the horizontal axis. Approximate curves are shown. (**B**) Relationship between CRST Part B drawing C scores and the amplitude of videographic parameters in a 15 cm line-drawing task. The CRST Part B drawing C evaluation scores are plotted on the vertical axis, with videographic amplitudes on the horizontal axis. Approximate curves are shown. (**C**) Relationship between the essential tremor rating assessment scale (TETRAS) 4b postural tremor scores and the amplitude of videographic parameters during the postural tremor task. TETRAS 4b postural tremor scores are on the vertical axis, with videographic amplitude on the horizontal axis. Approximate curves are shown. (**D**) Correlation between CRST Part A scores and logarithmic videographic amplitude during the postural tremor task. (**E**) Correlation between CRST Part B drawing C scores and logarithmic videographic amplitude in a 15 cm line-drawing task. (**F**) Correlation between TETRAS 4b postural tremor scores and logarithmic videographic amplitude during the postural tremor task.

Next, we aimed to address the ceiling effect in CRST evaluation through video motion analysis. In the postural tremor task, we investigated the relationship between the CRST Part A postural tremor score of the treated upper limb and the amplitude of the fingertip tremor measured by video analysis (Figure 2A). The results revealed that among the patients rated with four points on the CRST (indicating tremor amplitudes of 4 cm or more), three patients were identified, and one of them exhibited a tremor amplitude exceeding 10 cm (104 mm) in the video analysis. Similarly, one patient rated with three points (indicating tremor amplitudes between 2 and 4 cm) on the CRST demonstrated an amplitude of over 6 cm (69 mm) in the video analysis. Interestingly, some patients rated with two points on the CRST (indicating tremor amplitudes less than 2 cm) had amplitudes of 3 cm or more (36 mm).

Furthermore, we explored the relationship between the CRST Part B drawing C score in the 15 cm line-drawing task and the amplitude of the pen tip measured by video analysis (Figure 2B). The results highlighted the variability in the parameter values obtained from video analysis, even for patients with the same CRST score.

We examined the relationship between the TETRAS 4b postural tremor score (ranging from 0 to 4 points) of the treated upper limb and the amplitude of the fingertip tremor measured by video analysis (Figure 2C). While TETRAS demonstrates a clearer and more accurate quantification of essential tremor severity compared to CRST (Figure 2A), there was variability in the amplitude values from video analysis, even for patients with the same TETRAS score.

The regression analysis, correlating videographic amplitude with tremor scores as per clinical evaluation metrics (seen in Figure 2D–F), revealed a robust correlation between videographic amplitude and the clinically evaluated tremor scores. This correlation was particularly pronounced with TETRAS.

These findings suggest that video analysis has the potential to overcome the ceiling effect and address variability and limitations in CRST and TETRAS evaluations, thus underscoring the utility of video-based quantitative analyses.

### 3.5. Changes in Videographic Parameters by the MRgFUS Thalamotomy

Next, we compared data before and after MRgFUS thalamotomy to investigate whether videographic analysis could be used to assess treatment responsiveness (Table 5). MRgFUS thalamotomy showed significant improvements in all indicators of the CRST scores, except for CRST Part B on the non-treated side. Additionally, the video-graphic parameters showed significant improvements.

We further analyzed whether the degree of improvement in videographic analysis (logT2/T1) reflected the improvement in clinical indicators such as CRST scores and tremor scores (R2-R1) (Table 6). In the postural tremor task, the degree of improvement in fingertip amplitude was strongly correlated with the improvement in the tremor score, as well as the CRST Part B and Part C scores (*p* < 0.001). In the 15 cm line-drawing task, improvement in videographic amplitude showed significant correlations with improvement in the tremor score and CRST Part B score (*p* < 0.001).

## 4. Discussion

The use of 2D motion analysis software allowed us to successfully obtain the movement parameters of ET, including amplitude and cumulative length, from the video data of the postural tremor task and 15 cm line-drawing task. The videographic parameters demonstrated high reproducibility, effectively reflected clinical severity, overcame the ceiling effect observed in clinical indicators, and proved useful as evaluation tools for treatment efficacy. While amplitude is generally considered to impact tremor score, it is noteworthy that videographic amplitude values correlated with clinical indicators of ADL (CRST Part C). This finding suggests the importance of videographic amplitude values as a significant parameter reflecting aspects of ADL.

In the evaluation of the CRST using the 15 cm line-drawing task, it is typically assessed whether the lines drawn by patients intersect with the borders. This evaluation is believed to correlate with the amplitude of the video motion analysis parameters. Interestingly, in this study, we utilized the advantage of the 2D motion analysis software to track markers (pen tips) and measure the cumulative length of the pen tip movement. This allowed for the quantitative evaluation of severe tremor in the vertical direction that did not make contact with the paper but were projected onto the 2D plane. In this study, it was demonstrated that the cumulative length is strongly correlated not only with elements of tremor, such as CRST Part B and tremor score, but also with elements of ADL (Part C). This suggests the importance of accumulating further evidence regarding the usefulness of videographic cumulative tremor length.

CRST is limited in its use due to its ceiling effect [19]. As shown in Figure 2A, among the patients rated with “4 points” on the CRST, one individual had a video-based assessment exceeding 10 cm. When the tremor during the postural task exceeds 4 cm, all cases are evaluated as “4 points” on the CRST, indicating the presence of a ceiling effect. In contrast, the amplitude of finger tremor during the postural task obtained through video analysis ranged from 1.3 to 104.9 mm, showing a continuous range of values. This finding suggests that video analysis can overcome the ceiling effect when evaluating tremor.

Furthermore, in the 15 cm line-drawing task of the CRST, “4 points” is defined as the inability to complete the drawing. However, as demonstrated in Figure 2B, there is a significant variation in the distance required to draw a straight line of 15 cm among cases rated as “4 points”, indicating variability in amplitude. This suggests the presence of inter-rater variability in the CRST. In recent years, wearable devices have been used to address this variability among raters. Although the potential utility of these devices as tools for assessing treatment effectiveness during MRgFUS thalamotomy is intriguing, the video analysis examined in this study did not require marker placement during recording and allowed for the free selection of regions of interest for analysis after video capture. Considering that MRgFUS thalamotomy can treat various types of tremor in different scenarios, video analysis has the potential to be utilized for a more accurate assessment of tremor under various task conditions. While our findings indicate that videographic analysis provides a more nuanced measurement of tremor amplitude, it is important to consider its sensitivity to treatment effects relative to TETRAS. In our study, the MDC% for TETRAS and CRST did not show a significant difference, suggesting that while videography may offer more detailed amplitude measurements, its sensitivity to treatment effects compared to clinical ratings such as TETRAS is not substantially greater. This finding is in line with previous studies, such as the work of Elble and Ellenbogen [8], which have not demonstrated a definitive advantage of device-based measures over TETRAS in detecting treatment effects. Despite the potential advantages of videographic analysis in measuring tremor amplitude, further research is needed to fully establish its superiority over traditional clinical rating scales such as TETRAS in terms of sensitivity to treatment effects. Future studies should aim to include more direct comparisons and possibly incorporate the Weber–Fechner relationship in evaluating the sensitivity of these tools to changes post-treatment.

Despite these promising findings, our study had some limitations. First, it is a retrospective study, which may have inherent biases and limitations compared with prospective trials. Moreover, our dependence on 2D motion analysis software might have led to the underestimation of in-depth movements in our video analysis. This software is predominantly equipped for analyzing movements in horizontal and vertical planes. As a result, this methodological constraint could have hindered a complete capture and thorough evaluation of the depth component of the tremor movements in our study. Additionally, it is noted that the videographic tremor analysis was not conducted using spectral analysis. To achieve a more precise evaluation of tremor, it is imperative to develop algorithms for spectral analysis in future studies. Furthermore, because evaluation during the MRgFUS thalamotomy procedures was not performed in this study, it is necessary to conduct real-time video analysis during MRgFUS thalamotomy and provide feedback to the neurosurgeon to verify whether it contributes to improving treatment outcomes.

In conclusion, videographic motion analysis shows great promise as a valuable tool for the quantitative evaluation of ET and addresses the limitations of the existing clinical evaluation methods.

## Figures and Tables

**Table 1:** Patient Characteristics.

	Total	Reliability-Test Subgroup	*p* Value *
Number of patients, *n*	43	10	
Age, mean (SD)	69.9 (11.8)	68.8 (10.0)	0.144
Male sex, *n* (%)	29 (67.4)	8 (80.0)	0.876
Disease duration, years (SD)	27.7 (18.1)	27.9 (17.8)	0.437
Family history, *n* (%)	20 (46.5)	8 (80)	0.842
Baseline tremor severity, median (min-max)			
CRST total score	36.0 (10–76)	40.5 (14–70)	0.536
Part A score	7.0 (1–33)	10.0 (3–17)	0.093
Part B score, treatment side	10.0 (2–20)	11.5 (2–20)	0.089
Part B score, contralateral side	7.0 (0–16)	6.0 (1–14)	0.336
Part C score	10.0 (1–26)	11.0 (2–26)	0.286
Tremor score	14.0 (3–28)	14.5 (3–23)	0.145
Side of treatment, *n* (left:right)	37:6	10:0	

SD, standard deviation, * *p*-values were calculated using the *t*-tests.

**Table 2:** Test–retest reliability, minimal detectable change, and inter-rater reliability of the videographic parameters.

	Test–Retest Reliability		Inter-Rater Reliability
	ICC _(1,1)_ (95% CI)	MDC_95_	ICC _(2,3)_ (95% CI)
Postural tremor, fingertip			
Velocity	0.985 (0.943–0.996)	79.47 mm/s	0.990 (0.970–0.997)
Acceleration	0.999 (0.994–1.000)	726.81 mm/s^2^	0.996 (0.989–0.999)
Frequency	0.988 (0.967–0.997)	0.46 Hz	0.943 (0.833–0.985)
Amplitude	0.999 (0.997–1.000)	8.35 mm	0.998 (0.995–1.000)
15 cm Line-Drawing Task			
Cumulative length	0.999 (0.998–1.000)	24.91 mm	0.998 (0.996–1.000)
Velocity	0.998 (0.995–1.000)	40.65 mm/s	0.997 (0.991–0.999)
Acceleration	0.995 (0.986–0.999)	1991.94 mm/s^2^	0.995 (0.986–0.999)
Frequency	0.948 (0.802–0.987)	0.88 Hz	0.927 (0.787–0.980)
Amplitude	0.997 (0.988–0.999)	1.26 mm	0.997 (0.992–0.999)

ICC, intraclass correlation coefficient; CI, confidence interval; MDC_95_, 95% confidence interval of minimal detectable change.

**Table 3:** Minimum Detectable Change Results for CRST Part A, TETRAS, and Videographic-amplitude values.

Postural Tremor, Fingertip	CRST Part A			TETRAS			Video-Amplitude		
	Mean	Test 1–2 SDd	MDC%	Mean	Test 1–2 SDd	MDC%	Mean	Test 1–2 SDd	MDC%
Test 1	2.7	0.823	89%	1.75	0.408	84%	13.95	0.211	61%
Test 2	3.4			1.75			14.03		
15 cm Line-Drawing Task	CRST Part B						Video-amplitude		
	Mean	Test 1–2 SDd	MDC%				Mean	Test 1–2 SDd	MDC%
Test 1	1.2	−0.100	84%				5.16	0.056	51%
Test 2	1.3						5.12		

SDd, Standard Deviation of the Differences; MDC%, percentage of minimal detectable change. Mean: geometric mean (mm).

**Table 4:** Correlation between videographic parameters and clinical rating scores.

Videographic Parameters	CRST	CRST	CRST	CRST	CRST
(logT1)	Total score	Part A	Part B	Part C	Tremor score
Postural tremor, fingertip					
Amplitude	0.471 **	0.656 **	0.309 *	0.389 **	0.507 **
15 cm Line-Drawing Task					
Cumulative length	0.554 **	0.216	0.746 **	0.517 **	0.673 **
Amplitude	0.597 **	0.328 **	0.724 **	0.377 *	0.682 **

The values are Spearman’s Ro values. *: *p* < 0.05, **: *p* < 0.01.

**Table 5:** Changes in clinical rating scores and videographic parameters by the MRgFUS thalamotomy.

	Before MRgFUS	After MRgFUS	*p* Value *
CRST Score	median (min–max)	median (min–max)	
Total score	36.0 (10–76)	18.0 (2–48)	<0.001
Part A	7.0 (1–33)	4.0 (0–20)	<0.001
Part B treatment side	10.0 (2–20)	2.0 (0–10)	<0.001
Contralateral side	7.0 (0–16)	6.0 (0–16)	0.573
Part C	10.0 (1–26)	4.0 (0–13)	<0.001
Tremor score	14 (3–28)	2 (0–11)	<0.001
Videographic Parameters	mean (SD)	mean (SD)	
Postural tremor task, fingertip			
Amplitude (mm)	14.0 (19.7)	2.7 (3.2)	<0.001
15 cm line-drawing task, mean			
Cumulative length (mm)	414.5 (578.1)	159.0 (13.6)	<0.001
Amplitude (mm)	10.8 (15.0)	2.5 (5.1)	<0.001

* *p*-values were calculated using the Wilcoxon signed-rank test.

**Table 6:** Correlation between changes in videographic parameters and improvements of clinical indicators by the MRgFUS thalamotomy.

Logarithmic Changes (logT2/T1)	Tremor Score (R_2_ − R_1_)	Part B Score (R_2_ − R_1_)	Part C Score (R_2_ − R_1_)
Postural tremor, fingertip			
Amplitude	0.488 **	0.357 **	0.434 **
15 cm line-drawing task			
Cumulative length	0.606 **	0.683 **	0.398 **
Amplitude	0.577 **	0.587 **	0.191

The values of videographic parameters before MRgFUS thalamotomy were defined as T1, and the corresponding values after MRgFUS thalamotomy were defined as T2. The clinical scores before MRgFUS thalamotomy were defined as R1, and the corresponding scores after MRgFUS thalamotomy were defined as R2. The values are Spearman’s Ro values. **: *p* < 0.01.

## Data Availability

Data is contained within the article and supplementary material.

## Supplementary Materials

The following supporting information can be downloaded at: https://www.mdpi.com/article/10.3390/neurolint15040091/s1, Figure S1: The Fahn–Tolosa–Martin Clinical Rating Scale for Tremor (CRST); Figure S2: The illustrative picture of the postural tremor task; Figure S3: The illustrative picture of the 15 cm line-drawing task; Video S1: The illustrative movie of the postural tremor task; Video S2: The illustrative movie of the 15 cm line-drawing task.

## Abbreviations

CRST: Clinical Rating Scale for Tremor; FUS: Focused ultrasound thalamotomy; MRgFUS thalamotomy: Magnetic resonance-guided focused ultrasound thalamotomy; ET: Essential tremor; VIM: Ventral intermediate nucleus; SDR: Skull density ratio; ADL: Activities of daily living.
