# Supplementary material for: Video Motion Analysis as a Quantitative Evaluation Tool for Essential Tremor during Magnetic Resonance-Guided Focused Ultrasound Thalamotomy
Source: Neurol Int. 2023 Nov 29;15(4):1411–22. doi: 10.3390/neurolint15040091 (PMC10745303; doi:10.3390/neurolint15040091)
Supplement: Supplementary file 1 [file neurolint-15-00091-s001.zip › Supplementary Figures.pdf]

CLINICAL RATING SCALE FOR TREMOR

Right or Left Handed: Right□, Left□

Have you had caffeine during the past 8hrs? Yes□, No□

Have you had alchohol during the past 8hrs? Yes□, No□

Part A: Tremor Location/Severity Rating(1-10):

		Rest	Posture	Action/Intenton	Total
1	Face tremor	□0□1□2□3□4			
2	Tongue tremor	□0□1□2□3□4	□0□1□2□3□4		
3	Voice tremor			□0□1□2□3□4	
4	Head tremor		□0□1□2□3□4		
5	RUE tremor	□0□1□2□3□4	□0□1□2□3□4	□0□1□2□3□4	
6	LUE tremor	□0□1□2□3□4	□0□1□2□3□4	□0□1□2□3□4	
7	Trunk rremor		□0□1□2□3□4		
8	RLE tremor	□0□1□2□3□4	□0□1□2□3□4	□0□1□2□3□4	
9	LLE tremor	□0□1□2□3□4	□0□1□2□3□4	□0□1□2□3□4	
10	Orthostatic(trunk/legs when standing)		□0□1□2□3□4		

SUBTOTAL A:

PART B: Specific Motor Tasks/Functioning Rating(11-15):

		Right	Left	Total
11	Handwriting(Dominant only)	□0□1□2□3□4	□0□1□2□3□4	
12	Drawing A	□0□1□2□3□4	□0□1□2□3□4	
13	Drawing B	□0□1□2□3□4	□0□1□2□3□4	
14	Drawing C	□0□1□2□3□4	□0□1□2□3□4	
15	Pouring	□0□1□2□3□4	□0□1□2□3□4	

SUBTOTAL B:

Part C: Functional Disabilities Resulting from

16	Speaking	□0□1□2□3□4
17	Eating	□0□1□2□3□4
18	Drinking	□0□1□2□3□4
19	Hygiene	□0□1□2□3□4
20	Dressing	□0□1□2□3□4
21	Writing	□0□1□2□3□4
22	Working	□0□1□2□3□4
23	Social Activities	□0□1□2□3□4

SUBTOTAL C:

TOTAL SCORE:

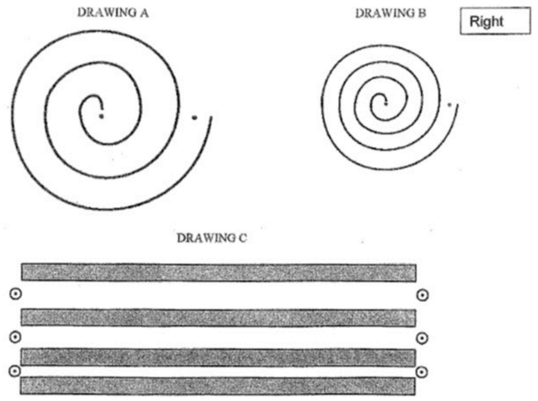

Suppliment Figure1. Clinical Rating Scale for Tremor (CRST)

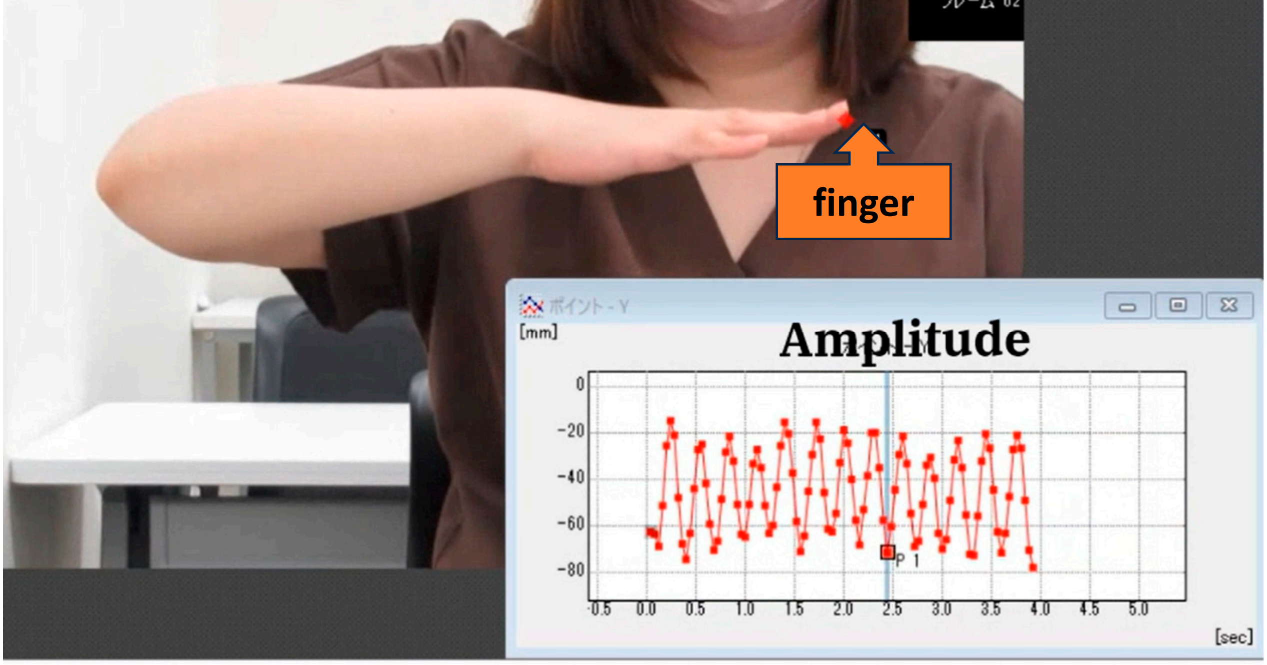

Supplement Figure 2. Videographic Evaluation of Postural Tremor

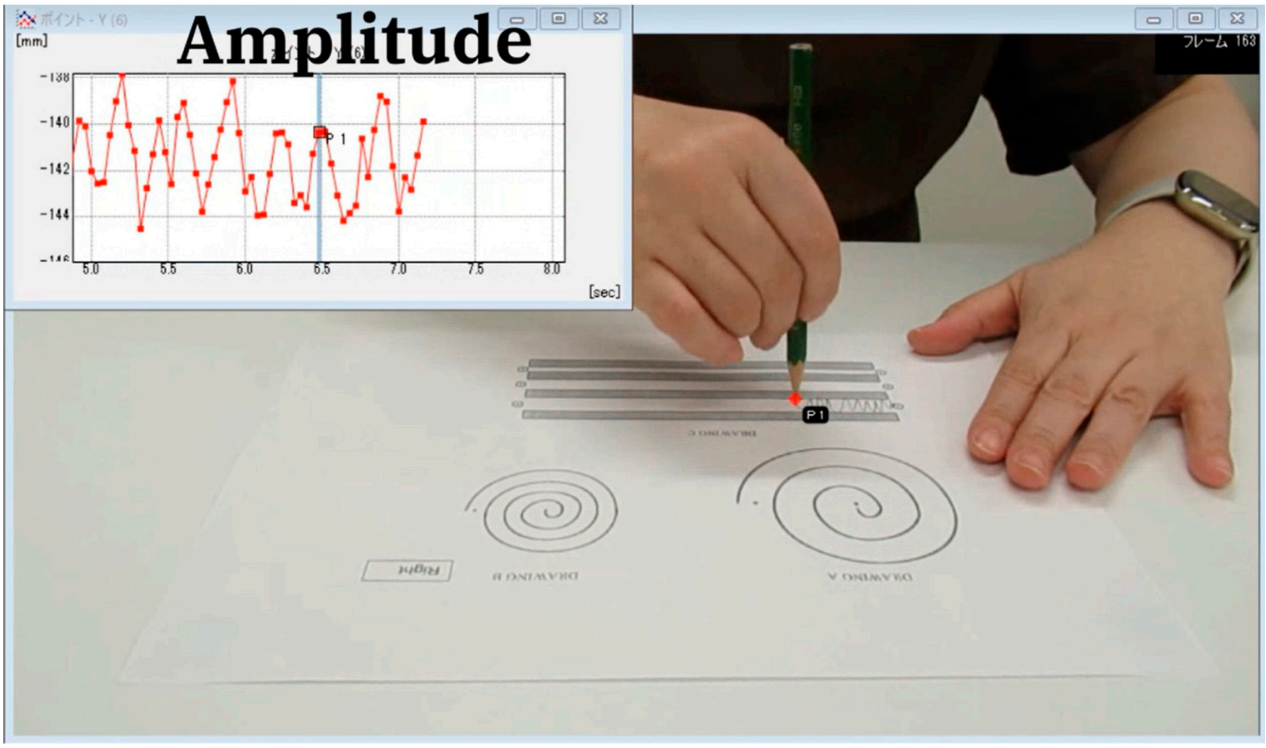

Supplement Figure 3. Videographic evaluation of the Line Drawing Task
